# The Relationship Between Psychological Empowerment and Clinical Decision-Making Among Staff Nurses in Governmental Hospital in Al-Baha, Saudi Arabia

**DOI:** 10.7759/cureus.56871

**Published:** 2024-03-25

**Authors:** Aisha Z Al-shomrani, Ghada M Hamouda, Nabellah Abdullah

**Affiliations:** 1 Nursing, King Abdulaziz University, Jeddah, SAU; 2 Nursing Administration, College of Nursing, King Abdulaziz University, Jeddah, SAU; 3 Public Health, King Abdulaziz University, Jeddah, SAU

**Keywords:** nurses’ decision-making, empowerment, nurses’ psychological empowerment, clinical decision-making, psychological empowerment

## Abstract

Background

The Saudi Arabian government has published its 2030 vision for improving health care to meet worldwide standards for the nursing profession. To fulfill this vision, building large-scale healthcare facilities is necessary. Among the most common occupations, nursing is vital to health care systems. Although working in health care institutions is challenging, demanding, and comprehensive, they are created to save lives and enhance patient satisfaction. Therefore, health care organizations must seek to develop psychologically empowered and decision-making nurses who can help meet clients’ demands and enhance patient care, safety, quality, and outcomes. This study aims to determine the association between psychological empowerment (PE) and clinical decision-making (CDM) among staff nurses.

Methods

This study employed a quantitative cross-sectional correlation design. Three Saudi Ministry of Health-affiliated hospitals in the Al-Baha region were included. The sample size was calculated using the Raosoft online sample size calculator, with a total of 318 participants. The study sample included nurses working in inpatient, outpatient, and critical care departments. Convenience sampling techniques with inclusion and exclusion criteria were employed. An online survey with three sections was used for data collection: sociodemographic characteristics, the psychological empowerment instrument, and the nursing decision-making instrument. Data collection began at the beginning of February 2023 and was completed by the beginning of April 2023.

Results

The participants were 318 nurses working in critical areas, inpatient, and outpatient departments at three governmental hospitals in the Al-Baha region. Overall, 285 participants (89.6%) had a high level of PE, and the majority, 263 participants (82.7%), exhibited flexible-oriented decision-making. Approximately three-quarters of the sample, 281 participants (88.4%), were female, and more than half of the staff nurses, 187 participants (58.8%), were married. The majority of participants, 250 (78.6%), had a bachelor's degree. Regarding professional experience, most staff nurses, 134 participants (42.1%), had between one and five years of experience, and the majority worked in inpatient units, 160 participants (50.3%), while 104 (32.7%) worked in critical care.

Conclusion

The current study found a significant association between nurses’ PE and CDM. Nurses with the highest PE were the most flexible in their CDM. Moreover, the findings of this study offer some points that nurse managers and leaders can use to generate empowerment and make their staff better decision-makers. One recommendation is to develop training and coaching programs to enhance PE among staff nurses, thereby raising their work meaningfulness, which would reflect in better CDM. Additionally, this study recommends that future research be conducted to examine how PE affects CDM.

## Introduction

The world is undergoing a significant knowledge and technological revolution, which poses some difficulties for health development worldwide. The Saudi Arabian government has published its 2030 vision for improving health care to meet the worldwide standards for the nursing profession [1]. To achieve this vision, it is necessary to build large-scale health care facilities. Among the most common occupations, nursing is vital to health care systems. Therefore, health care organizations must seek to develop psychologically empowered decision-makers who can help meet patients’ demands and enhance patient care, safety, quality, and outcomes. The nursing profession is dealing with constant challenges due to the dynamic demands and vigorous evolution of the health care environment. Working in an environment of uncertainty, disempowerment, and high organizational needs stresses nurses, jeopardizing their physical and emotional well-being and the nursing profession itself. Empowered nurses who can make clinical decisions will improve nursing autonomy and patient outcomes, which is critical for health care organizations.

Regarding leadership, enhancing employee empowerment will be helpful during times of uncertainty. Nurse executives must build supportive settings that promote employee control and moderate the negative impact of rapid changes in health care. Leaders must be self-assured and in a positive emotional state to support others. They also need great levels of psychological empowerment (PE) to meet challenges in the work environment. Spreitzer [2] explored the concept of empowerment and described PE as a type of intrinsic motivation based on four perceptions: competence, meaning, self-determination, and impact. It is stated that the lack of any one of these four characteristics reduces the overall degree of seen empowerment and that these four components comprise the entire PE concept.

According to Ghalavi and Nastiezaie [3], PE begins with changes in the staff’s attitudes, ideas, and beliefs. In other words, individuals need to feel confident in their capacity to carry out their duties effectively and their power to shape and direct the course of their careers. Emerging research suggests that leaders should attempt to foster PE because it is crucial to improve the quality of nursing care. Leaders’ empowering behaviors seem essential in developing a professional work environment and raising the standards of nursing care [4,5]. Many exploratory studies that use PE as an independent variable have revealed that the degree of personal PE has some bearing on outcome variables such as satisfaction, productivity, work behavior, work attitude, burnout, and performance. For example, a cross-sectional study by Mudallal et al. [4] emphasized the significance of the role of nurse leaders in enhancing work environments and empowering and inspiring nurses to lower burnout, lower turnover rates, and enhance the standard of nursing care.

Moreover, PE encourages employees to work to the best of their abilities. It is a crucial component of clinical decision-making (CDM) and particularly essential for a company’s success, among many other factors. Furthermore, it fosters involvement in CDM and in creating and implementing innovative ideas. Employee PE and CDM are critical to an employee’s functioning because when poor decisions are made, they are a sign of an underlying issue and a leading cause of anticipated difficulties in the organization. Nursing commitment and patient care will improve if staff nurses can engage in all levels of CDM. It is crucial to comprehend the complex nature of professional autonomy to design appealing workplaces. Nurses must be allowed to participate in CDM and develop their nursing skills through shared leadership [6]. Additionally, patient care quality and safety are improved by empowered decision-maker nurses, which will reflect on staff morale, productivity, staff retention, and associated costs. In addition, empowering nurses in decision-making will increase their ability to make critical decisions, which improves workplace satisfaction, nursing autonomy, and patient outcomes.

In nursing practice, enhancing nurses’ cognitive abilities is necessary for CDM and intuition. This enables them to choose the best nursing alternatives for their patients and to directly and indirectly identify the problems affecting patients [7]. A nurse’s CDM refers to their intuitive ability to facilitate a healthy life for the patient. Lauri and Salanterä [8] proposed the CDM models. They include an analytical model, an intuitive model, an intuitive-analytical model, and an analytical-intuitive model. According to Anton et al. [9], nurses can assess complex clinical situations; provide a fast and confident assessment; and provide patients with safe, high-quality care using intuitive or subconscious decision-making processes based on their extensive expertise.

Indeed, decision-making is crucial to all managerial and human activity, but its processes are frequently complicated. It is necessary to comprehend what elements can slow them down. It's not easy to make the best clinical decision. The nurse may be diverted from making the best decision by a variety of variables, such as nursing level of education, assistant-in-nursing skills, knowledge and attributes, organizational and unit culture, comprehension of patient status, situation awareness, and autonomy [10,11]. Moreover, because the work environment directly affects a nurse’s CDM, organizational and cultural factors may promote or restrict their decision-making ability.

Generally, nurses’ decisions significantly affect patient safety and care quality outcomes. Furthermore, the severity of patients’ illnesses, external pressure, and personal stress may have a negative impact on nurses’ decision-making [12]. However, making the wrong decisions is critical because this will affect individual and organizational performance and employees’ futures. In this regard, nurses should first set priorities and choose which patients to help when dealing with several patient requirements.

Moreover, PE is logically an important element of CDM, and it is crucial in creating a professional practice environment and improving the quality of nursing care. However, to the best of our knowledge, few studies have been conducted in Saudi Arabia investigating the relationship between PE and CDM among staff nurses. Because of this, a knowledge gap exists about how these variables are related. Therefore, the current study fills national and international research gaps by providing valuable evidence on the relationship between PE and CDM in nursing. It will also contribute to the development of the nursing field, including the efficiency of organizations and clinical practice. The aim of this study is to fill the gap and determine whether a relationship exists between PE and CDM among staff nurses.

Conceptual framework

The conceptual framework played a crucial role in guiding the researcher throughout the study. This study aims to determine the relationship between PE and clinical decision-making among staff nurses in a government hospital. The conceptual framework was developed based on Spreitzer's [2] and Lauri and Salanterä's [8] instruments. This framework will guide the research in understanding the complex relationship between PE and clinical decision-making among staff nurses in a governmental hospital.

In aspects of PE and its dimensions, Spreitzer [2] proposed a multidimensional construct comprising four dimensions: meaning, competence, self-determination, and impact. Meaning refers to employees' understanding of the significance of their work, aligning with the meaningfulness aspect of clinical decision-making, where nurses find purpose in their actions to improve patient outcomes. Competence reflects the perception of one's skills and capabilities, akin to the proficiency required for effective clinical decision-making. Self-determination emphasizes the autonomy and discretion individuals have in performing their roles, paralleling nurses' autonomy in making clinical decisions based on their expertise. Finally, impact refers to individuals' perception that they can influence outcomes, akin to the nurses' belief in the influence of their decisions on patient care.

For clinical decision-making and its influences, Lauri and Salanterä [8] proposed a model incorporating both cognitive and intuitive processes. Nurses rely on clinical expertise, knowledge, and evidence-based practices to make informed decisions. This aligns with the "competence" dimension of PE, where nurses' perceived competence positively influences their ability to make sound clinical decisions. Moreover, nurses' self-determination to act independently and their belief in the impact of their decisions are key aspects influencing clinical decision-making. Nurses who feel empowered are more likely to exhibit initiative, engage in shared decision-making with colleagues, and advocate for optimal patient care. This sense of autonomy and influence aligns with the self-determination and impact dimensions of PE.

As shown in the literature discussion above, some studies have explored the relationship between PE and clinical decision-making among nurses. Empowered nurses tend to be more proactive in seeking information, collaborating with interdisciplinary teams, and critically evaluating various options before making decisions. Their belief in their competencies and autonomy equips them to make well-informed, patient-centered choices.

Based on these foundations, the researcher establishes the study concept, which is PE, referring to the sense of control, autonomy, and self-efficacy that staff nurses feel in their work environment. Clinical decision-making is the process through which staff nurses assess patient needs, identify appropriate interventions, and make critical choices regarding patient care.

There were four categories of variables identified based on this concept. (1) The independent variable is PE; this is the main factor that we believe will influence clinical decision-making among staff nurses. (2) The dependent variable is clinical decision-making, the outcome that is affected by PE. (3) Mediating variables include self-efficacy, which is the belief in one's abilities to perform clinical tasks and make sound decisions; job satisfaction, which is the level of contentment and fulfillment experienced by nurses in their work; and perceived support, which is the perception of support from supervisors and the organization in the decision-making process. (4) Moderating variables are the experience level, indicating that the amount of experience nurses have in their role may impact the relationship between PE and clinical decision-making, and organizational culture, suggesting that the culture of the hospital and its support for empowerment may influence decision-making.

The conceptual framework stated above explains the expected outcomes of this study. The conceptual framework developed based on Spreitzer’s (1995) and Lauri and Salantera’s (2002) tools provides a robust foundation for examining the intricate interplay between PE and clinical decision-making. Further empirical research is needed to explore this relationship more comprehensively, taking into account contextual factors and potential moderating variables within the healthcare setting.

This study expects to find a positive relationship between PE and clinical decision-making among staff nurses. Mediating variables like self-efficacy, job satisfaction, and perceived support are expected to explain the mechanism behind this relationship. Finally, moderating variables such as experience level and organizational culture are anticipated to influence the strength of the relationship.

## Materials and methods

Aim and design

The study employed a quantitative, cross-sectional correlation design to investigate the relationship between PE and CDM among staff nurses, assess staff nurses’ level of PE, and examine the level of CDM among staff nurses.

Setting and sample

This study was conducted in three government hospitals in the Al-Baha region of Saudi Arabia. The study population included inpatient, outpatient, and critical care department nurses. Convenience sampling was adopted. An adequate sample size was required to answer the research question; a smaller sample size might impair the results’ dependability. The Raosoft online sample size formula was used to calculate the sample size (http://www.raosoft.com/samplesize.html). The total number of participants was 318. All registered nurses who worked in the inpatient, outpatient, and critical care departments were included. Nurses who worked in other clinical areas, such as administrative areas, and nurses under training (interns and students) were not included. Purposeful sampling with a nonprobability sampling technique was employed.

Data collection and instruments

The researcher used two self-administered online questionnaires to collect data from participants about CDM and PE. Additionally, meetings with the director of nursing were held in each hospital to introduce the researcher and discuss the study’s goals. The researcher created the sociodemographic characteristics section, which included age, gender, nationality, marital status, education level, years of experience, working hospital, and unit or department. The first tool used was Spreitzer’s [2] PE instrument. The researcher obtained permission to use the questionnaire based on Spreitzer’s work. It comprised 12 items, divided into four categories: meaning (three items), competence (three items), self-determination (three items), and impact (three items). Participants responded using a five-point Likert scale (1 = strongly disagree, 2 = disagree, 3 = not sure, 4 = agree, and 5 = strongly agree). The researcher determined the overall mean for all 12 items. The minimum and maximum overall scores were 12 and 60, respectively, for each of the four dimensions. A score between 12 and 24 indicated a poor perception of PE; a score between 25 and 36 indicated a moderate perception; and a score between 37 and 60 indicated a high perception of PE. 

The second tool, adopted from the study by Lauri and Salantera [8], collected information about CDM in nursing. Permission was obtained to use a questionnaire based on Lauri and Salantera’s work. It comprised 24 items. Participants responded using a five-point Likert scale (1 = almost never, 2 = rarely, 3 = sometimes, 4 = often, and 5 = almost always). Scores for responses to odd items were reversed. For example, if the respondent selected response option 1, that response would be scored as 5; option 2 would be scored as 4; option 3 would remain unchanged; option 4 would be scored as 2; and option 5 would be scored as 1. Scores for even items were not changed. The scores were added together, and the total was interpreted as follows: a mean score of >78 indicated the staff nurse had intuitive decision-making capability; a mean score between 68 and 78 indicated flexible decision-making capability; and a mean score of <67 indicated analytically oriented decision-making capability.

Participants could respond to the online questionnaires by using the provided link, which was available for two months (February 1, 2023, to April 1, 2023). The duration of the study was one year, beginning in January 2023 and ending in January 2024. The researcher provided their phone number and email address to enable participants to ask questions about the study.

Validity and reliability of the tools

Both questionnaires were presented to a professional jury of assistant and associate professors from King Abdul-Aziz University’s nursing faculty. Before the questionnaires were delivered to the participants, experts with prior knowledge of and experience in the study topic reviewed their contents to ensure they were valid, consistent, and clear. The jury members noted that the assertions in the questionnaires were reasonable, pertinent, and consistent with the study’s goal. They offered feedback and evaluated the questionnaires’ appropriateness, determining that no adjustments were necessary. A pilot study was conducted with 31 staff nurses from the initial sample (10% of the population) to gather input regarding the questionnaires’ structure, readability, relevance, design, and the time needed to complete them. The validity of a questionnaire, which is the extent to which it reflects the concept being studied, was assessed. For the study domains of PE and CDM, Cronbach’s alpha coefficients were excellent (0.96 and 0.89, respectively).

Data analysis

The data extracted were encoded in an Excel sheet (Microsoft Corporation, Redmond, Washington), and the participants' names were numerically encoded to maintain anonymity. The data were then analyzed using IBM SPSS Statistics for Windows, Version 29 (Released 2022; IBM Corp., Armonk, New York). Data entry for demographic variables was conducted using a numerical code for each variable listed in the questionnaire. The mean and standard deviation for each item in the PE and CDM sections of the study sample were calculated using a one-sample t-test. A mean score and a p-value are considered significant if the p-value is less than 0.05; if a p-value is greater than 0.05, then the result is deemed insignificant. The relationship between demographic data and the overall scores for CDM tools and PE was analyzed using one-way ANOVA. A p-value of less than 0.05 is generally accepted as indicating statistical significance. Additionally, a chi-square correlation test was conducted to examine the association between the research participants' PE and CDM. The association was considered significant if its p-value was less than 0.05. To facilitate the analysis of the data, graphs and tables were employed.

Ethical considerations

To conduct a study, the researcher must adhere to all ethical standards. The Research Ethics Committee of King Abdulaziz University's nursing department granted ethical permission (Ref. No. 2M.49). Additionally, in August 2022, ethical consent was obtained from the Ethics Research Committee of Directors of Health Affairs in the Al-Baha region of Saudi Arabia (No. 23-08-2022/3). Before filling out the questionnaire, participants gave their informed consent. The researcher explained the study's primary goal, procedures, and significance. To avoid bias during data collection, the author knew none of the participants. All completed questionnaires were discarded after data analysis.

## Results

Demographic data

Table 1 reveals that the number of female nurses was higher than that of male nurses (88.4%), and most were in the 30-40-year age group. Saudis comprised only 27.7% of staff nurses, whereas non-Saudis comprised the majority of staff nurses (72.3%). Regarding educational attainment, 78.6% of nurses had a bachelor's degree. Regarding marital status, more than half of the staff nurses were married (58.8%). Regarding hospitals, 34.9% of nurses worked at Prince Mashari Hospital, followed by Al-Mikhwah General Hospital (34.0%) and King Fahad Hospital (31.1%). Regarding participants' professional experience, most staff nurses had between one and five years of experience (42.1%), and most worked in inpatient units (50.3%).

**Table 1:** Distribution of sociodemographic characteristics of staff nurses in this study (n=318)

Variables	Categories	N	%
Gender	Male	37	11.6
	Female	281	88.4
Nationality	Saudi	88	27.7
	Non-Saudi	230	72.3
Age	20 to <30 years	107	33.6
	30 to <40 years	154	48.4
	40 to <50 years	39	12.3
	≥50 years	18	5.7
Education	Diploma	41	12.9
	Bachelor’s degree	250	78.6
	Master’s degree	22	6.9
	Doctoral degree	5	1.6
Marital status	Single	114	35.8
	Married	187	58.8
	Divorced	15	4.7
	Widowed	2	0.6
Working hospital	King Fahad Hospital	99	31.1
	Prince Mashari Hospital	111	34.9
	Al-Mikhwa General Hospital	108	34
Years of experience at the current hospital	Less than 1 year	41	12.9
	1 year to <5 years	134	42.1
	5 years to <10 years	58	18.2
	10 years and more	85	26.7
Working unit	Critical care units	104	32.7
	Inpatient units	160	50.3
	Outpatient units	54	17

Tables 2, 3 show the level of PE. Most participants, i.e., 285 (89.6%), showed a high level of PE, 26 (8.2%) were at a moderate level, and seven (2.2%) exhibited poor PE.

**Table 2:** The mean and standard deviation calculated for psychological empowerment items in the study sample (n = 318) using a one-sample t-test The mean score and *p*-value are significant if <0.05 using a one-sample *t*-test

Item		Strongly disagree	Disagree	Not sure	Agree	Strongly agree	Mean	Median	SD	P-value
Meaning							12.242	12	2.429	0.000
Item 1	N	11	12	21	161	113	4.11	4	0.94	0.000
	%	3.5%	3.8%	6.6%	50.6%	35.5%				
Item 2	N	14	8	30	179	87	4	4	0.93	0.000
	%	4.4%	2.5%	9.4%	56.3%	27.4%				
Item 3	N	10	11	21	160	116	4.14	4	0.91	0.000
	%	3.1%	3.5%	6.6%	50.3%	36.5%				
Self-determination							11.494	12	2.475	0.000
Item 4	N	10	18	35	177	78	3.93	4	0.93	0.000
	%	3.1%	5.7%	11.0%	55.7%	24.5%				
Item 5	N	13	27	33	176	69	3.82	4	1	0.000
	%	4.1%	8.5%	10.4%	55.3%	21.7%				
Item 6	N	13	30	39	179	57	3.75	4	0.99	0.000
	%	4.1%	9.4%	12.3%	56.3%	17.9%				
Competencies							12.355	12	2.373	0.000
Item 7	N	10	8	25	149	126	4.17	4	0.91	0.000
	%	3.1%	2.5%	7.9%	46.9%	39.6%				
Item 8	N	12	9	19	167	111	4.12	4	0.92	0.000
	%	3.8%	2.8%	6.0%	52.5%	34.9%				
Item 9	N	10	6	30	180	92	4.06	4	0.86	0.000
	%	3.1%	1.9%	9.4%	56.6%	28.9%				
Impact							11.214	12	2.419	0.000
Item 10	N	15	12	59	180	52	3.76	4	0.93	0.000
	%	4.7%	3.8%	18.6%	56.6%	16.4%				
Item 11	N	10	22	60	179	47	3.73	4	0.91	0.000
	%	3.1%	6.9%	18.9%	56.3%	14.8%				
Item 12	N	12	18	63	177	48	3.73	4	0.92	0.000
	%	3.8%	5.7%	19.8%	55.7%	15.1%				
Total							47.305	48	8.303	

**Table 3:** The level of psychological empowerment in the study sample (n = 318)

Psychological empowerment	Score	N	%	Mean ± SD
Poor	12–24	7	2.2%	47.305±8.303
Moderate	25–36	26	8.2%	
High	37–60	285	89.6%	
Total	12-60	318	100%	

Tables 4, 5 show the level of CDM. The results showed that the mean score for the CDM scale total was (mean = 70.79, SD = 3.56), indicating that the CDM level of nurses was generally flexible, with 263 (82.7%) being flexible, 49 (15.4%) analytically oriented, and six (1.9%) intuitively oriented.

**Table 4:** The mean and standard deviation calculated for clinical decision-making items in the study sample (n = 318) using a one-sample t-test The mean score and *p*-value are significant if <0.05 using a one-sample *t*-test.

Item		Option					Mean	Median	SD	P-value
		Never or almost never	Rarely	Not rarely or not often	Often	Almost always or always				
Item 1	N	8	36	39	127	108	3.92	4	1.07	0.000
	%	2.5%	11.3%	12.3%	39.9%	34.0%				
Item 2	N	26	51	51	125	65	3.48	4	1.21	0.000
	%	8.2%	16.0%	16.0%	39.3%	20.4%				
Item 3	N	13	26	42	147	90	3.86	4	1.05	0.000
	%	4.1%	8.2%	13.2%	46.2%	28.3%				
Item 4	N	15	41	67	128	67	3.60	4	1.10	0.000
	%	4.7%	12.9%	21.1%	40.3%	21.1%				
Item 5	N	9	26	64	151	68	3.76	4	0.97	0.000
	%	2.8%	8.2%	20.1%	47.5%	21.4%				
Item 6	N	8	29	51	152	78	3.83	4	0.98	0.000
	%	2.5%	9.1%	16.0%	47.8%	24.5%				
Item 7	N	13	22	55	142	86	3.84	4	1.03	0.000
	%	4.1%	6.9%	17.3%	44.7%	27.0%				
Item 8	N	11	24	45	152	86	3.78	4	1	0.000
	%	3.5%	7.5%	14.2%	47.8%	27.0%				
Item 9	N	12	17	49	150	90	3.91	4	0.99	0.000
	%	3.8%	5.3%	15.4%	47.2%	28.3%				
Item 10	N	17	41	58	142	60	3.59	4	1.10	0.000
	%	5.3%	12.9%	18.2%	44.7%	18.9%				
Item 11	N	5	25	39	155	94	3.97	4	0.94	0.000
	%	1.6%	7.9%	12.3%	48.7%	29.6%				
Item 12	N	6	30	59	142	81	3.82	4	0.98	0.000
	%	1.9%	9.4%	18.6%	44.7%	25.5%				
Item 13	N	5	22	42	153	96	3.98	4	0.92	0.000
	%	1.6%	6.9%	13.2%	48.1%	30.2%				
Item 14	N	12	24	50	164	68	3.79	4	0.99	0.000
	%	3.8%	7.5%	15.7%	51.6%	21.4%				
Item 15	N	9	19	50	154	86	3.91	4	0.96	0.000
	%	2.8%	6.0%	15.7%	48.4%	27.0%				
Item 16	N	5	11	56	136	110	4.05	4	0.90	0.000
	%	1.6%	3.5%	17.6%	42.8%	34.6%				
Item 17	N	6	16	53	141	102	4	4	0.93	0.000
	%	1.9%	5.0%	16.7%	44.3%	32.1%				
Item 18	N	5	14	42	149	108	4.07	4	0.89	0.000
	%	1.6%	4.4%	13.2%	46.9%	34.0%				
Item 19	N	5	17	43	143	110	4.06	4	0.91	0.000
	%	1.6%	5.3%	13.5%	45.0%	34.6%				
Item 20	N	12	19	48	154	85	3.88	4	0.99	0.000
	%	3.8%	6.0%	15.1%	48.4%	26.7%				
Item 21	N	4	19	45	147	103	4.03	4	0.91	0.000
	%	1.3%	6.0%	14.2%	46.2%	32.4%				
Item 22	N	7	19	48	149	95	3.96	4	0.94	0.000
	%	2.2%	6.0%	15.1%	46.9%	29.9%				
Item 23	N	7	18	52	149	92	3.95	4	0.94	0.000
	%	2.2%	5.7%	16.4%	46.9%	28.9%				
Item 24	N	5	13	54	149	97	4.01	4	0.88	0.000
	%	1.6%	4.1%	17.0%	46.9%	30.5%				
Clinical decision-making							70.79	71	3.56	

**Table 5:** The level of nursing clinical decision-making in the study sample (n = 318)

Clinical decision-making	Score	N	%	Mean ± SD
Analytically oriented	Less than 67	49	15.4%	70.79±3.56
Flexible	67 - 78	263	82.7%	
Intuitively oriented	More than 78	6	1.9%	
Total	33-120.	318	100%	

Table 6 shows a significant association between PE and CDM, where the p-value was <0.05.

**Table 6:** A chi-square correlation test was used to calculate the correlation between psychological empowerment and clinical decision-making The p-values are significant if <0.05 using a chi-square correlation test.

Level		Decision-making is analytically oriented	Decision-making is flexible	Decision-making is intuitively oriented	Total	Chi-square (p-value)
Poor empowerment	N	0	7	0	7	15.677 (0.003)
	%	0%	2.2%	0%	2.2%	
Moderate empowerment	N	4	19	3	26	
	%	1.3%	6%	0.9%	8.2%	
High empowerment	N	45	237	3	285	
	%	14.2%	74.5%	0.9%	89.6%	
Total	N	49	263	6	318	
	%	15.4%	82.7%	1.9%	100%	

## Discussion

Psychological empowerment

This study demonstrated that nurses highly valued PE and believed it to be effectively defined by its competence, meaning, impact, and self-determination elements. Consistent with the results in [13], the results showed that nurses felt competent, had sufficient influence to affect organizational outcomes, and found meaning in their work. The results of the present study showed that the participants had a high mean score for PE overall (mean±SD, 47.305±8.303). This result confirms that there are few barriers in the workplace that prevent employees from addressing issues affecting their day-to-day work and that the management of Al-Baha hospitals offers a comparatively high degree of PE. The results were also consistent with the study by Abu Hashish et al. [14], who reported that the level of PE was high. Competence was the highest-ranking dimension, followed by meaning, self-determination, and impact. Furthermore, in contrast to our findings, prior research indicated variations within participant groups regarding PE rankings, with certain groups scoring highest in impact, while other groups scored lowest [15]. The current study showed that nurses’ PE had a high mean score. The study conducted in Saudi Arabia by [16] examined nurses’ PE in pediatric units and found that PE was high, supporting our findings. A further rationale for the high level of PE experienced by nurses was that the majority of them held bachelor's degrees in nursing and were in their thirties or older.

In contrast, a cross-sectional study conducted by Asiri et al. [17], who investigated the relationships among leadership style, PE, and organizational commitment in Saudi Arabia among staff nurses. The findings indicated that nurses’ PE was at a moderate level. Furthermore, the study by Saleh et al. [18] revealed low levels of PE among nurses. In the current study, the association between PE and demographic data was the second variable. The most notable finding from the current research was the significant relationship between PE and nationality. The current study revealed that registered non-national nurses were more psychologically empowered than Saudi nurses. This aligned with the findings of Alharbi and Alrwaitey [16], who showed that non-Saudi nurses were more psychologically empowered than Saudi nurses. The reason for this result was the variation in the number of national and non-national participants. Moreover, previous studies evaluating Saudi nurses’ working environments found that they experienced stressful workplaces, ambivalence, and dissatisfaction with their professions [19]. They also found a significant association between PE and education. This association was stronger among participants with a bachelor's degree. This result was inconsistent with a study conducted in Iran [20], which reported significant differences between PE and educational level. Technical nurses demonstrated higher levels of PE compared to those with bachelor’s and postgraduate degrees. This study found no significant association between age and PE, in contrast to a survey conducted in China [21], which found a significant association between age and PE. Furthermore, our findings revealed no significant link between PE and years of experience.

Clinical decision-making

In the context of a health organization, CDM is a sophisticated cognitive process that nurses use to recognize clinical problems in their patients and take prompt action to improve the patient’s rapidly and regularly shifting health status [22]. The present study’s findings revealed that the mean score of nurses’ CDM was (mean = 70.79, SD = 3.56). The findings demonstrated nurses’ three decision-making abilities: intuitive, flexible (analytical-intuitive), and analytical. The intuitive interpretive and analytical systematic models (intuitive and analytical approaches) primarily explain nurse decision-making. The analytical approach consists of logical reasoning that is systematic and requires a review of all the data obtained thus far. According to the intuitive interpretive model, experts make decisions based on intuitive principles [23].

This study showed that nurses were flexibly oriented in their CDM level (82.7%). Consistent with the findings of Kosicka et al. [24], this study’s findings revealed that most participants exhibited flexibility in their CDM. In contrast to the results of Abate et al. [23], the surveys in this study indicated that 55.7% of nurses used analytical decision-making, whereas 44.3% used intuitive decision-making. Moreover, a study conducted in Saudi Arabia [25] found that most participants were intuitive in their CDM. Research by Lauri and Salantera conducted in the United States, Canada, Finland, and Norway verified that nurses had different decision-making levels based on their practice areas. Finnish nurses made analytical clinical decisions, but Canadian community-family nurses made intuitive ones. American and Norwegian nurses used analytical-intuitive reasoning to make decisions [24,26].

This study found that certain factors influenced nurses’ CDM regarding demographic data. The results mostly showed that age, education, and years of experience were significantly related to nurses’ decision-making, while gender, marital status, working unit, and nationality were not associated with decision-making. The findings showed a statistically significant relationship between participants’ decision-making and age, consistent with the findings of Bakalis et al. [27], who argued that increased age indicates that nurses make more decisions about unit budgets and organize others’ work. Concerning clinical experience, the findings showed that experience level influenced decision-making. They supported the findings of Farčić et al. [28], who demonstrated that nurses’ perceptions of their CDM were more positive when they had greater job experience. In this light, clinical experience is a crucial factor and an essential predictor of decision-making. This research revealed that educational level impacts decisions. The expectation of having the flexibility to choose what to do and how to do it increases with one’s level of qualification. These findings were corroborated by Wu et al. [29], whose cross-sectional nonexperimental study demonstrated that nurses’ CDM abilities greatly improved based on their level of education.

The relationship between nurses’ PE and CDM

To determine the relationship between PE and decision-making, we examined whether there was a connection between these two variables (Table 6). All p-values were less than 0.05. This research discovered a significant association between PE and CDM. Nurses with the highest PE were the most flexible in CDM. For instance, in the context of inappropriate management of decision-making, Zeng et al. conducted a multicenter cross-sectional study and suggested that PE was positively associated with CDM [30]. This indicates that nursing staff can make decisions and manage their affairs to pursue significant and worthwhile career objectives.

The study’s findings demonstrated that PE had positive effects on nurses’ CDM. Employees were more likely to make adaptable therapeutic decisions when they experienced a high level of PE at work. Conversely, employees with lower PE made fewer clinical decisions.

Limitations

By conducting this study, we acknowledge its strengths, such as the high level of PE reported by participants and the significant association found between PE and CDM. However, we also recognize its limitations. A cross-sectional study design was applied, which cannot establish a cause-and-effect relationship or analyze behavior over time. A longitudinal study through a nursing school and practice would better describe and explain the relationship between PE and CDM over time. Moreover, the study was conducted in hospitals in the Al-Baha region, and only the nursing staff provided data for this study, which may impact the findings' applicability to all Saudi Arabian hospitals and may limit their generalizability. Additionally, we used a non-probability sampling technique during data collection, which may have led to sampling bias. Future studies are encouraged to use larger and more representative samples for all the analyzed variables to improve the generalizability of the findings. Therefore, future research might replicate and extend the scope of the study, especially in different work environments-that is, different health care facilities, different settings, and different populations.

Recommendations

The results from this research may help managers and organizations recognize how to enhance employees' CDM through PE, as our findings reveal that employees' decision-making is linked to their level of PE. For instance, creating a workplace that encourages nurses to participate in decision-making is critical. Additionally, by making resources and information easily accessible, hospitals can foster psychological stability in their workforce, leading to better decision-making. Furthermore, in the practice field, the findings of the research might help guide and enhance clinical nursing practice in several ways: the establishment of structured programs to strengthen PE among staff nurses, the implementation of training and coaching programs to develop and raise their work meaningfulness, and the creation of a monitoring system to gauge the progress of employee performance. Future research may explore how PE affects CDM further. It is also recommended that future researchers employ other sampling approaches to obtain larger data samples and improve the study's generalizability.

## Conclusions

The research yielded significant findings regarding PE, CDM, and the relationship between these two variables. The results indicate that participants exhibited a high overall level of PE, with competence ranking as the highest and impact as the lowest. In terms of CDM, the findings revealed that nursing decision-making was predominantly flexible (82.7%). A significant association was observed between nurses’ PE and their CDM capabilities. Nurses with the highest levels of PE demonstrated the greatest flexibility in CDM.

## Figures and Tables

**Figure 1:**
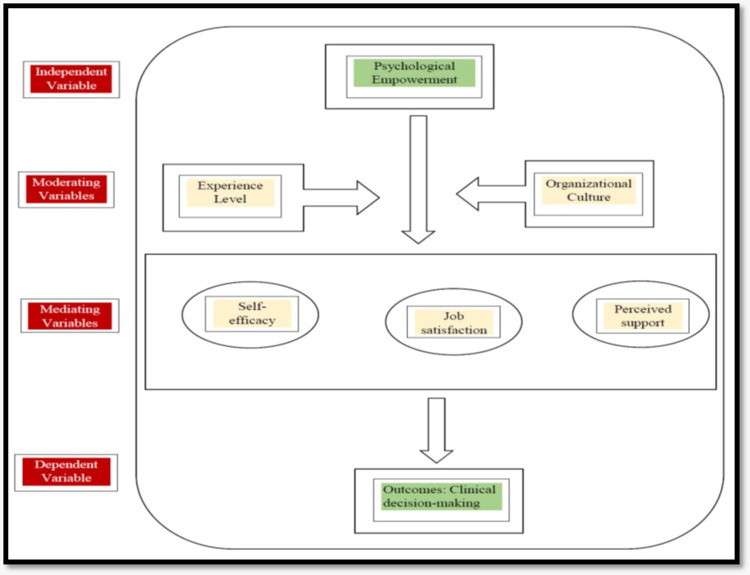
Conceptual framework created based on Spreitzer (1995) and Lauri & Salantera (2002)

## Appendices

**Table 7:** Psychological empowerment instrument

Items	Strongly disagree (1)	Disagree (2)	Not sure (3)	Agree (4)	Strongly agree (5)
(1) Meaning					
The work I do is meaningful to me.					
My job activities are personally meaningful to me.					
The work that I do is important to me.					
Self-determination					
I have significant autonomy in determining how I do my job.					
I can decide on my own how to go about doing my own work.					
I have considerable opportunities for independence and freedom in how I do my job.					
Competencies					
I am confident about my ability to do my job					
I am self-assured about my capabilities to perform my work activities.					
I have mastered the skills necessary for my job.					
Impact					
My impact on what happens in my department is large.					
I have a great deal of control over what happens in my department.					
I have significant influence over what happens in my department.					

**Table 8:** Nursing decision-making instrument

Items	Never or almost never (1)	Rarely (2)	Not rarely or not often (3)	Often (4)	Almost always or always (5)
1. I collect information from the patient’s records.					
2. I rely on my own interpretations.					
3. I specify all the items I intend to monitor and ask the patient about.					
4. I make assumptions during the first contact with the patient.					
5. I confirm the impression on the advance information that supports my views.					
6. It is easy for me to make a distinction in defining the patient’s condition.					
7. I compare the information I have with my earlier knowledge.					
8. I compare the information I have with my own experiences.					
9. I compare the information I have with research knowledge.					
10. It is easy for me to see, which pieces of information are relevant.					
11. I define the patient’s nursing problems objectively.					

Items	Never or almost never	Rarely	Not rarely or not often	Often	Almost always or always
12. It is easy for me to form an overall picture of the patient’s situation.					
13. I draw up the patient’s nursing plan according to the nursing decision-making process.					
14. I base the patient’s nursing plan on my own nursing views.					
15. I base the patient’s nursing plan on the patient's disease.					
16. I document without difficulties the general patient’s care to the patient's records.					
17. I set out targets for the patient’s care.					
18. I anticipate the nursing interventions on the patient.					
19. I follow closely the patient’s existing nursing plan.					
20. I anticipate changes in the patient’s condition.					
21. I use specific information about the treatment of the patient’s disease.					
22. I flexibly change my line of action.					
23. I try to find reasons for changes in the patient’s condition.					
24. It is easy for me to assess my actions on the patient’s condition.					
